# Benzylidene Cyclopentanone Derivative Photoinitiator for Two-Photon Photopolymerization-Photochemistry and 3D Structures Fabrication for X-ray Application

**DOI:** 10.3390/polym15010071

**Published:** 2022-12-24

**Authors:** Anton E. Egorov, Alexey A. Kostyukov, Denis A. Shcherbakov, Danila A. Kolymagin, Dmytro A. Chubich, Rilond P. Matital, Maxim V. Arsenyev, Ivan D. Burtsev, Mikhail G. Mestergazi, Elnara R. Zhiganshina, Sergey A. Chesnokov, Alexei G. Vitukhnovsky, Vladimir A. Kuzmin

**Affiliations:** 1 N. M. Emanuel Institute of Biochemical Physics, Russian Academy of Sciences, 119334 Moscow, Russia; 2 Moscow Institute of Physics and Technology, National Research University, Institutskii per. 9, 141700 Dolgoprudny, Russia; 3 G. A. Razuvaev Institute of Organometallic Chemistry, Russian Academy of Sciences, 603950 Nizhny Novgorod, Russia; 4 Department of Medicinal Chemistry, Ernest Mario School of Pharmacy, Rutgers, The State University of New Jersey, Piscataway, NJ 08854, USA; 5 Lebedev Physical Institute, Russian Academy of Sciences, Leninskii Prospect 53, 119991 Moscow, Russia

**Keywords:** benzylidene ketone, photoinitiator, triplet state, two-photon photopolymerization (TTP), direct laser writing photolithography (DLW photolithography), X-ray microlenses

## Abstract

Micron- and submicron-scale 3D structure realization nowadays is possible due to the two-photon photopolymerization (TPP) direct laser writing photolithography (DLW photolithography) method. However, the achievement of lithographic features with dimensions less than 100 nm is in demand for the fabrication of micro-optical elements with high curvature values, including X-ray microlenses. Spectroscopic and photochemical study of a photoinitiator (PI) based on a methyl methacrylate derivative of 2,5-bis(4-(dimethylamino)benzylidene) cyclopentanone was performed. Enhanced intersystem crossing in the methyl methacrylate derivative results in increased radical generation for the subsequent initiation of polymerization. A comprehensive study of the new photocompositions was performed, with particular emphasis on photochemical constants, the degree of photopolymerization, and topology. The optimal parameters for the fabrication of mechanically stable structures were determined in this research. The threshold dose parameters for lithography (radiation power of 5 mW at a speed of 180 µm/s) when trying to reach saturation values with a conversion degree of (35 ± 1) % were defined, as well as parameters for sub-100 nm feature fabrication. Moreover, the 45 nm feature size for elements was reached. Fabrication of X-ray lens microstructures was also demonstrated.

## 1. Introduction

Two-photon photopolymerization (TPP, DLW photolithography) is a unique technology that allows for the fabrication of polymeric 3D structures with the required submicron spatial resolution in terms of their morphology and topology. This technology is based on two-photon absorption of a focused femtosecond laser radiation by a photosensitive component (photoinitiator), which initiates the polymerization reaction. As a result, a polymerized element is formed in the focal region. Because two-photon absorption probability is proportional to the squared intensity of light, the excitation volume is confined mostly to the focal spot. Due to this peculiarity, TPP makes it possible to diminish voxel (a 3D analogue of a pixel in photography) size, an advantage compared to one-photon polymerization processes. Moreover, the use of NIR femtosecond laser excitation sources allows for increased penetration depth since most of the polymers are near transparent in this spectral region [1,2]. Due to the possibility of achieving submicron elements for 3D structures, DLW photolithography and two-photon absorption techniques have found application in such areas as the development of photonic crystals [3,4], microrobotics and metamaterials [5,6,7], biomedicine [8], microfluidics research [9,10], micro-optics [11,12,13,14,15], hydrogel nanoarchitecture [16], and photovoltaics [17]. Recently, the possibility of fabricating X-ray microlenses using DLW photolithography was demonstrated. Such structures allow for X-ray radiation to be focused at a distance of 120 mm (numerical aperture NA = 1.7 × 10^−4^) into a spot with a diameter of less than 500 nm [18]. The advantage of such an approach is that a microlens system with an arbitrary curvature radius can be fabricated using DLW photolithography. It is possible to improve the quality of such structures through investigation of the material’s properties after the pyrolysis process [19]. However, further research is required to reduce voxel size in order to achieve an increase in the numerical aperture of these objects.

Photoinitiators based on benzylidene ketone moiety are promising for photocomposition preparation [20]. Single-component photoinitiation systems greatly simplify preparation. The bis-benzylidene ketone carcass is an example of a D-π-A-π-D system where electron donors and accepting groups are bridged with a π-conjugated chain. By changing the length of the π-conjugated chain of the dye, one can shift the absorption band, thus varying the depth of light penetration and enabling visible light polymerization. This capability, along with intensive molar extinction (up to 1 × 10^5^ M^−1^ cm^−1^), makes these dyes versatile in the photoinitiation process. The molecule possesses both electron-withdrawing (carbonyl) and electron donor (amino) groups, resulting in a charge transfer process. Bis-benzylidene ketone dyes possess the ability to initiate both one- and two-photon polymerization processes [20]. These PIs undergo photobleaching during the photopolymerization process [21]. This feature could be beneficial because no decrease in photopolymerization process speed due to absorption of the dye takes place. The facile synthesis process of these compounds is another trait that makes these PIs attractive.

**Figure 1:**
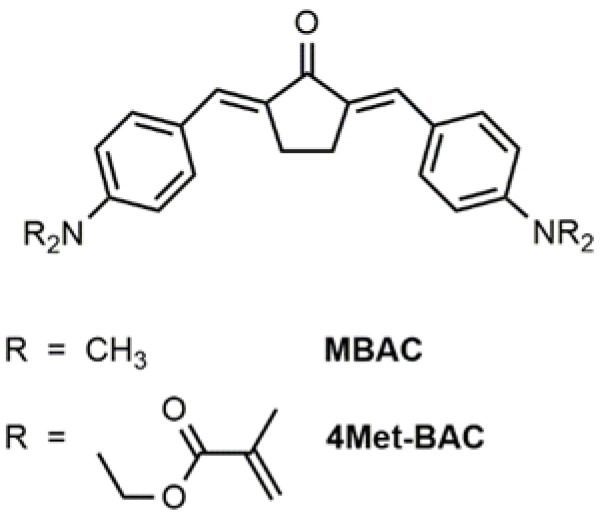
Molecular structure of MBAC and 4Met-BAC photoinitiators.

In this paper, the photochemistry of bis-(dimethylaminoarylidene) cyclopentanone containing four methyl methacrylate groups (4Met-BAC) was compared to the widely known molecule 2,5-bis(4-(dimethylamino)benzylidene) cyclopentanone (MBAC) and was investigated (Figure 1). The two-photon polymerization process was demonstrated using a 4Met-BAC PI. The introduction of methyl methacrylate groups into the PI molecule allows for increased solubility of the PI in monomer due to the chemical similarity that is gained. A comprehensive study of PI photochemistry and the mechanical and morphological properties of 3D nanosized structures made it possible to determine the optimal characteristics of new photocompositions.

## 2. Materials and Methods

### 2.1. Materials

Solvents for spectroscopic studies were spectroscopic grade (Komponent-Reaktiv, Moscow, Russia), and others were purified by the standard methods [22]. The monomer pentaerythritol triacrylate (PETA) (“Aldrich”) was used without additional purification. MBAC was synthesized by the well-known procedure [23]. (4-Formylphenyl)azandiyl)bis(ethane-2,1-diyl)dibenzoate was prepared according to the procedure described in [24].

### 2.2. Synthesis of 4Met-BAC

((((1E,1′E)-(2-oxocyclopentane-1,3-diylidene)bis(methanylylidene))–bis(4,1-phenylene)) -bis(azanetriyl))tetrakis(ethane-2,1-diyl) tetra-kis(2-methylacrylate) (4Met-BAC). To a suspension of benzylidenecyclopentanone tetraol (2.33 g, 5 mmol) and K_2_CO_3_ (10.4 g, 7.5 mmol) in dichloromethane (100 mL), a solution of methacryloyl chloride (2.05 mL, 21 mmol) in dichloromethane (20 mL) was added dropwise with cooling. The mixture was stirred for 24 h and the precipitate was filtered. Hexane (30 mL) was added to the filtrate and the product was crystallized, filtered, and dried in vacuo. Orange-red fine crystalline powder with a yield of 2.66 g (72%) was obtained. Elements found: C, 69.95; H, 6.89; N, 3.71. Calc. for C_43_H_50_N2O_9_: C, 69.90; H, 6.82; N, 3.79 %. ^1^H-NMR (300 MHz, CDCl_3_), δ (ppm): 1.93 (s, 12H, CH_3_(meth)), 3.06 (s, 4H, CH_2_ (c-pentanone)), 3.75 (t, 8H, OCH_2_, J = 6.2 Hz), 4.35 (t, 8H, NCH_2_, J = 6.2 Hz), 5.59 (s, 4H, C_Met_H), 6.09 (s, 4H, C_Met_H), 6.84 (d, 4H, C_Ar_, J = 8.9 Hz), 7.52 (s, 2H, OCC=CH), 7.54 (d, 4H, C_Ar_, J = 8.9 Hz). ^13^C-NMR (75 MHz, CDCl_3_), δ (ppm): 196.11, 167.30, 148.00, 135.89, 134.01, 133.26, 132.82, 126.20, 125.12, 111.97, 61.54, 49.43, 26.62, 18.36. IR (Nujol, ν/cm^−1^): 1375, 1457, 1522, 1590, 1678, 1715, 2927, 3400 (Figures S1 and S2).

### 2.3. Absorption Spectroscopy

UV–vis absorption spectra were recorded on a Shimadzu UV-3101 PC spectrophotometer in the range from 350 to 700 nm in quartz cells with an optical pathlength of 1 cm, unless otherwise noted.

### 2.4. Fluorescence Spectroscopy

Fluorescence measurements and singlet oxygen luminescence experiments were performed with a FluoTime 300 fluorescence lifetime spectrometer (PicoQuant GmbH, Berlin, Germany). For fluorescence quantum yield determination experiments, compound solutions of matched optical density (<0.05 per cm) were used at an excitation wavelength of 491 nm. Fluorescence lifetime measurements were made through picosecond time-correlated single-photon counting (TCSPC) and a pulsed laser light source (475 nm). Emission data were collected at 600 nm. The instrument response function (IRF) was recorded with the use of a Ludox probe. For singlet oxygen luminescence detection experiments, a spectrometer equipped with the NIR PMT Module H10330-45 (Hamamatsu, Japan) coupled to the single-photon counter TimeHarp TCSPC (PicoQuant Gmbh, Berlin, Germany) was used. Singlet oxygen luminescence experiments were performed using a quartz cuvette with a 1 cm optical path. The excitation source used was a Xe lamp (438–470 nm).

### 2.5. Flash Photolysis

The transient absorption spectra of radical intermediates were measured using a conventional flash photolysis setup (LLC “MELZ”, Moscow, Russia) (optical path length 20 cm, excitation performed through multi-band blue-green optical absorption filters 420–550 nm, 80 J/15 µs). Signals were recorded using a PMT-38 photomultiplier (LLC “MELZ”, Moscow, Russia) at 400–600 nm.

### 2.6. Laser Flash Photolysis

Laser pulse photolysis was carried out on an LKS 80 (Applied Photophysics, Leatherhead, UK) laser pulse photolysis setup. The third harmonics of a Nd-YAG laser (Brilliant B, Quantel, LUMIBIRD, Cournon-d’Auvergne, France) was used for excitation. Excitation wavelength in the range 420–600 nm was tuned by an OPO (MagicPrism, OPOTEK Inc., Carlsbad, CA, USA). Kinetics of transient species generated by a 5 ns laser excitation pulse were registered by difference absorbance changes in the spectral range of 400–750 nm using a 150 Xe arc lamp with 50-fold beam overdrive and 1.5 ms capacitor discharge. The detection system was equipped with a 600 MHz oscilloscope (Agilent Infiniium 10 GS/s) and an R928-type PMT. The kinetic data were processed through global analysis by fitting the kinetic traces over the whole range of the registration wavelengths with a multiexponential equation (Equation (1)):

ΔA_λ_ = ∑ΔA_λi_exp(−t/τ_i_) (1)

where ∆A_λ_ is the overall difference absorbance at the registration wavelength λ, ∆A_λi_ is the absorbance of the i-th transient species at the registration wavelength λ, and τ_i_ = 1/*k*_i_ is the lifetime of the i-th transient species, with τ_i_ and ∆A_λi_ being the fitting parameters. The accuracy in the lifetime determination was 15%.

### 2.7. DLW Photolithography

In this research, polymer structures were fabricated using DLW photolithography. The fabrication was carried out with a commercial setup (Nanoscribe Professional GmbH, Nanoscribe, Eggenstein-Leopoldshafen, Germany). The initiating two-photon photopolymerization reaction involved femtosecond laser radiation with an 80 MHz repetition rate and a 780 nm wavelength λ. This radiation was focused with a Zeiss high-aperture planapochromatic objective (NA = 1.4, 63). A new photosensitive composition based on the photoinitiator 4Met-BAC with methyl groups (1% wt) was used as a negative photoresin. Samples were developed in propylene glycol monomethyl ether acetate (PEGMA) for 25 min and then in isopropyl alcohol (IPA) for 5 min after the DLW lithography process.

### 2.8. Morphology

The influence of DLW photolithography parameters on the obtained voxel lithography element’s physical properties was investigated. The atomic force microscopy method (AFM, Solver Pro M, NT-MDT) was employed to study the axial size of a voxel. Measurements were carried out in a semicontact scanning mode. Scanning electron microscopy (SEM JEOL JSM-7001F, MIPT CUC, JEOL Ltd., Tokyo, Japan) was used to characterize the smallest axial size of the polymerized elements. A sample was prepared for morphological study. This sample contained arrays of linear voxel elements which were made with different output laser radiation powers. Within the array, the lithography linear velocity was varied to change the exposure time.

### 2.9. Confocal Laser Scanning Microscopy (CLSM)

Commercial Confocal Laser Scanning Microscopy (CLSM510, Carl Zeiss, Jena, Germany) was employed for the examination of the fabricated 3D polymer X-ray microlenses. Measurement of the 3D image along the z-axis was carried out using a scan mode Z-stack, with an excitation laser at 458 nm and a 40×/1.3 (Plan-Neofluar 40×/1.3, Carl Zeiss, Jena, Germany) oil immersion objective lens. Detection of the luminescence signal of the 3D polymer structure was carried out at the wavelength of 488 nm using a spectral long pass filter with a transmission wavelength of 505 nm and photomultiplier tubes which were equipped in the microscope.

### 2.10. Raman Spectroscopy

The spectroscopic properties of the polymerized and non-polymerized photocomposition were investigated using Raman spectroscopy. The measurements were carried out using a Horiba JY LabRAM HR Evolution Raman spectroscopy setup (Horiba, Kyoto, Japan). The laser wavelength was 633 nm, average laser power was 3.5 mW, and the grating was 1800 grooves/mm. One Raman spectrum was recorded with an exposure time of 70 s and averaged over 2 acquisitions.

## 3. Results

### 3.1. Spectroscopic Studies

#### 3.1.1. Absorption and Fluorescence Spectroscopy

**Figure 2:**
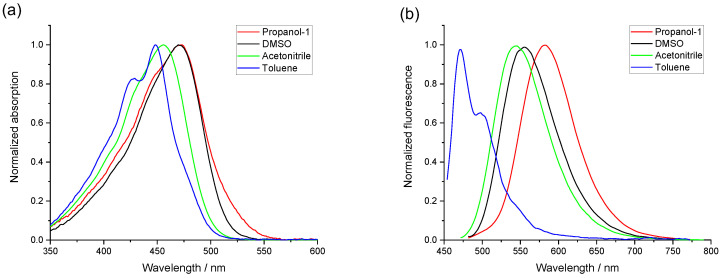
(**a**) Normalized 4Met-BAC absorption spectra in different solvents. (**b**) Normalized 4Met-BAC fluorescence spectra in different solvents.

Having an electron-donating amino group and electron-accepting carbonyl group results in charge transfer in photoinitiator molecules. Due to intramolecular charge transfer of the excited-state dyes, substantial positive solvatochromism could be observed (Figure 2a,b, Table 1). With the increase in solvent polarity, bathochromic shift of both absorption and emission spectra took place, confirming that S_1_ is of (π, π*) type [25].

In nonpolar aprotic solvents such as toluene, the fluorescence quantum yield decreases with the introduction of methacrylate groups into ketocyanine dye molecules (Table 2). The reason for this is the partial disruption of molecule planarity and the increase in vibrational relaxation processes. In protic polar solvent such as propanol-1, the excited-state dye is stabilized by both hydrogen bonding and dipole–dipole interaction. The red shift in both absorption and fluorescence spectra is the most pronounced in propanol-1, despite the relatively low dielectric constant compared to DMSO. This is due to lowering of the excited energy levels in solvent–solute complexes.

**Figure 3:**
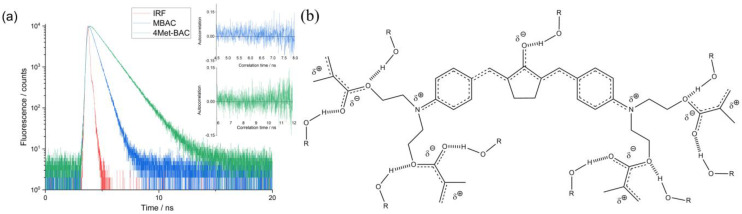
(**a**) Fluorescence kinetics decay of MBAC (blue) and 4Met-BAC (green) in propanol; excitation 475 nm. (**b**) Proposed hydrogen bonding of 4Met-BAC molecules in alcohol solvent.

Electronic interactions between polar solvent molecules and polar solute play an important role in stabilization of the excited state. In the case of protic solvents, hydrogen bonding also takes place. These effects could be observed in fluorescence lifetime measurements (Table 2). The most short-lived MBAC and 4-Met-BAC fluorescence belongs to the toluene solution (in highly polar acetonitrile it is almost three times larger). An interesting observation is made for propanol-1 solution. While 4Met-BAC and MBAC fluorescence lifetime is slightly different among nonprotic solvents, in propanol-1 solution the distinction is more prominent (0.46 ns vs. 1.16 ns) (Figure 3a), which also affects the difference between fluorescence quantum yields as well.

Alcohol hydrogen bonding ability towards the ketocyanine dye carbonyl group was previously established [26]. 4Met-BAC bears not only the central carbonyl group, but also the four methacrylic ester groups. The latter are capable of hydrogen bonding with alcohol molecules (Figure 3b). Thus, the multiplication of a hydrogen bond number in the case of 4Met-BAC results in amplified fluorophore stabilization and increases in fluorescence lifetime.

Spectral shifts with the increase in solvent polarity are more pronounced for fluorescence than absorption. This indicates that the excited state dipole moment is larger than ground state one.

#### 3.1.2. Excited State Dipole Moment

Intramolecular charge transfer results in reorientation of solvent molecules surrounding the solute [27]. The excited state dipole moment was calculated according to Bakhshiev’s formula [28]:

(2) $$v_{a}-v_{f}=\frac{2\Delta \mu^{2}}{a_{0}^{3}hc}F$$

where,

(3) $$F=\left(\frac{D-1}{D+2}-\frac{n^{2}-1}{n^{2}+2}\right)\cdot \frac{\left(2n^{2}+1\right)}{\left(n^{2}+2\right)}$$

where Δ*μ*^2^ = (*μ*_e_ − *μ*_g_)^2^, *υ_a_* and *υ_f_* are absorption and emission maxima wavenumbers, *μ*_e_ and *μ*_g_ are the excited and ground singlet state dipole moment, respectively, *a*_0_ is the solute cavity radius, *h* is Planck’s constant, *c* is the speed of light, and *D* and *n* are the relative permittivity and refractive index, respectively.

**Figure 4:**
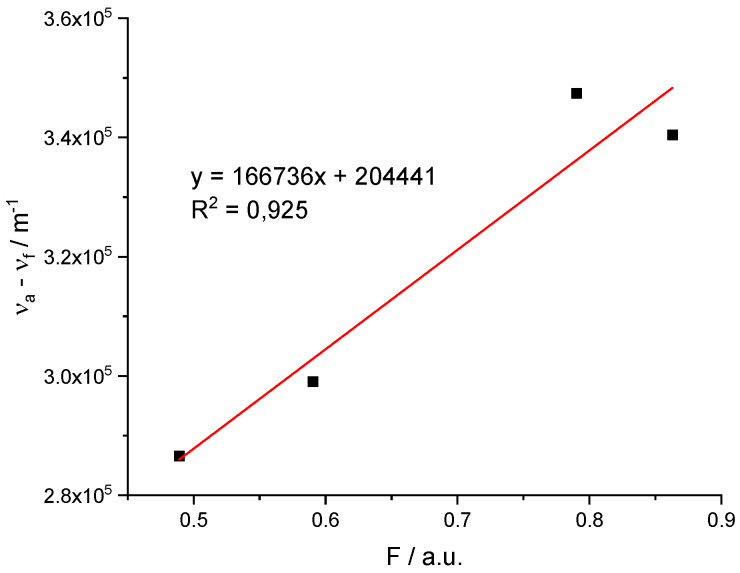
Plot between solvent spectral shifts and F solvent term for 4Met-BAC.

The plot of Stokes shift versus F (Figure 4) yields a slope from which *μ*_e_ can be calculated:

(4) $$Slope=\frac{2{\left(\mu_{e}-\mu_{g}\right)}^{2}}{a_{0}^{3}hc}$$

Protic and nonpolar solvents were excluded since a different kind of interaction takes place.

The solute cavity radius for 4Met-BAC was estimated according to [29] using the density of benzophenone. The ground state dipole moment was calculated using Hyperchem 8.0 software. Semiempirical calculations utilizing the INDO method to optimize molecular geometry resulted in 4.2 D and 4.5 D for 4Met-BAC and MBAC molecules, respectively, which is in accordance with [30,31]. The excited state dipole moment was estimated to be 10.8 D for 4Met-BAC, while for non-substituted MBAC it was estimated by another group to be 10.4 D [30]. The results show no substantial change in excited state dipole moment upon introduction of four methacrylic ester groups since the electron-donating ability of substituents is close.

#### 3.1.3. HOMO-LUMO

**Figure 5:**
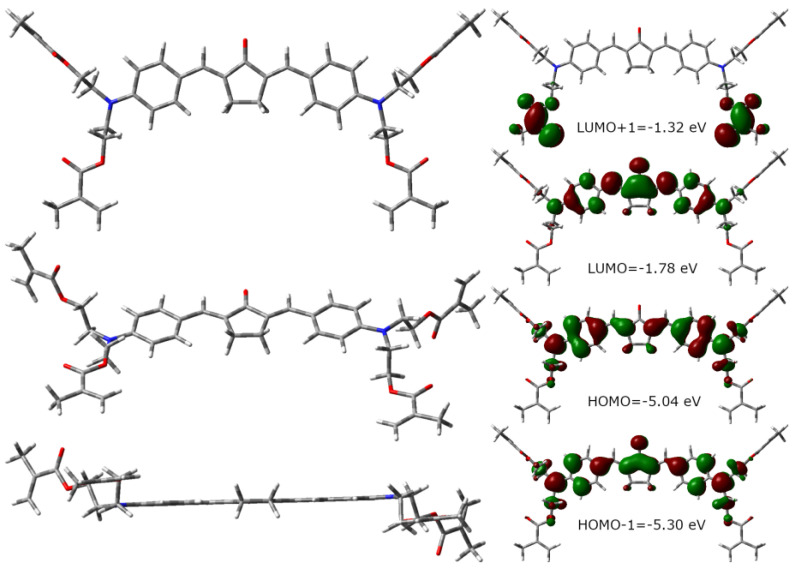
Optimized structure and molecular orbitals of 4Met-BAC.

Quantum chemical calculation of 4Met-BAC was performed using the ORCA 4.0.1.2 program package and density functional theory at the B3LYP functional with a 6-31G(d) basis set. 4Met-BAC’s structure has a planar geometry excluding peripheral methyl methacrylate substituents (Figure 5).

The HOMO and HOMO-1 orbitals have π symmetry, with the electrons distributed through the whole conjugated π system. The HOMO π electron shows an interruption of the conjugation at the carbonyl group. LUMO level is mainly located at carbonyl and cyclopentanone groups. These electron density distributions indicate that HOMO ⇒ LUMO transitions comprise intermolecular charge transfer.

#### 3.1.4. Triplet State Investigation

**Figure 6:**
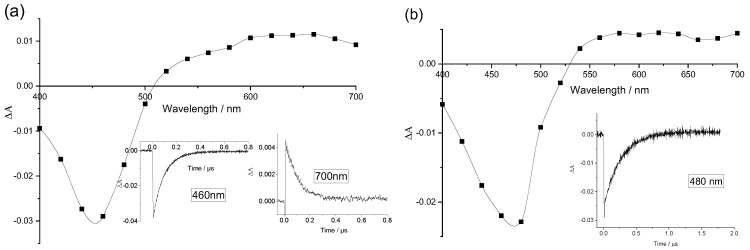
(**a**) Differential triplet–triplet absorption spectrum for 4Met-BAC (8.0 × 10^−6^ M) in acetonitrile (air-equilibrated) at 35 ns after flash. Excitation at 455 nm. Inserts show photobleaching of the singlet state at 460 nm and decay of the triplet state of 4Met-BAC at 700 nm (*k* = 1.1 × 10^7^ s^−1^). (**b**) Differential triplet–triplet absorption spectrum for 4Met-BAC (8.0 × 10^−6^ M) in propanol-1 (air-equilibrated) at 50 ns after flash. Excitation at 475 nm. Insert shows 4Met-BAC singlet state photobleaching at 480 nm (*k* = 3.8 × 10^6^ s^−1^).

Laser flash photolysis experiments were conducted with 4Met-BAC in different solvents. In all cases, triplet–triplet absorption spectra include photobleaching of the singlet state, with position corresponding to the singlet absorption spectra. Longer wavelength regions demonstrate triplet absorption that gradually intensifies with the increasing wavelength (Figure 6a,b).

In order to estimate triplet generation efficiency in every solvent, 4Met-BAC solutions with equal optical density at the excitation wavelength were prepared. Obtained triplet absorption maxima were compared in toluene, acetonitrile, and propanol-1. Triplet state generation ability is the most prominent in acetonitrile, closely followed by toluene; in the case of propanol-1 it is around 2.5 times less compared to acetonitrile. These results agree with the triplet state quantum yield determination experiments for MBAC derivatives [31]. The presence of carbonyl groups introduces excited (n, π*) terms into energetic level systems of molecules. The El-Sayed rule states [32] that intersystem crossing rate between excited states of different types exceeds that of the same type of excited states. Excited state energy level position depends on the solvent. For the similar ketocyanine dye, it was determined that the ^1^(π, π*) energy level lies above both ^3^(n, π*) and ^3^CT energy levels in nonpolar solvents (cyclohexane, toluene). In the case of polar solvents, ^1^(π, π*) lies above ^3^CT but below the ^3^(n, π*) energy level [25,31].

For closely related ketocyanine dye, the charge transfer state was established to be the lowest singlet state [31,33]. In nonpolar solvents such as toluene, the ^1^CT state lies above both ^3^(n, π*) and ^3^CT states, thus following the El-Sayed rule where ISC to ^3^(n, π*) first takes place and then IC quickly changes to the lowest ^3^CT state. However, due to relatively close proximity between the ^3^(n, π*) and ^3^CT state, the thermal population of ^3^(n, π*) occurs. This explains the high radical formation activity in toluene. In propanol-1, the ^1^CT state lies above ^3^CT but below ^3^(n, π*). Since ISC between ^1^CT and ^3^(n, π*) deals with the increase in energy, it is inefficient. Moreover, the transition between ^1^CT and ^3^CT is hindered, since the same type of excited states are involved. The energy gap between ^3^CT and ^3^(n, π*) is wider, so the thermal population role decreases. This explains both lowered triplet state generation and decreased reactivity towards radical formation. Since the ordering of energy levels is the same in both acetonitrile and propanol-1, the probable explanation for high triplet generation efficiency in the case of the former solvent is the position of these levels: the ^3^(n, π*) state lies much closer to the ^3^CT state, which makes is easier for thermal population of ^3^(n, π*) to take place.

Due to the charge–transfer nature of the 4Met-BAC triplet state in propanol, it is easily stabilized by protic solvent molecules. In the case of acetonitrile, the triplet state is ^3^(n, π*) in nature and is less influenced by solvent molecules. The values of triplet state oxygen quenching rate constants (Table 3) were calculated according to following equation:

*k* = *k*_T_ + *k*_q_[O_2_] (5)

where *k*_T_ is the triplet decay rate constant in the absence of oxygen (s^−1^) (Equation (6)) and *k*_q_ is the triplet state oxygen quenching rate constant (M^−1^s^−1^) (Equation (7)).

4Met-BAC(T_1_) ⇒ 4Met-BAC(S_0_) (6)

Calculation of the diffusional rate constant was performed using solvent viscosity according to [34]. The oxygen concentration values for different solvents were obtained according to [35,36]. The *k*_q_ values obtained for acetonitrile and propanol were close to *k*_diff_ multiplied by a spin-statistical factor of 1/9 (Table 3). These results are similar to the ones obtained in [31].

#### 3.1.5. Singlet Oxygen Quantum Yield

The ability to generate singlet oxygen is related to intersystem crossing processes in photoinitiator molecules. Singlet oxygen forms as a result of energy transfer between the photoinitiator triplet state and molecular oxygen (Equation (7)):

4Met-BAC(T_1_) +^3^O_2_ ⇒ 4Met-BAC(S_0_) +^1^O_2_    *k*_q_ (7)

^1^O_2_ ⇒ ^3^O_2_ + hυ (8)

**Figure 7:**
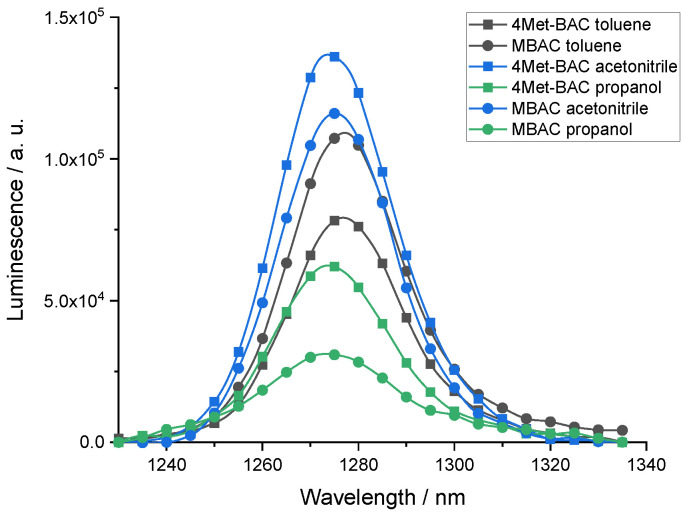
Singlet oxygen luminescence spectra of 4Met-BAC (6.0 × 10^−6^ M) (-■-) and MBAC (6.0 × 10^−6^ M) (-●-) solutions in different solvents. Excitation at 438–470 nm using a Xe lamp.

The luminescence of singlet oxygen could be registered at ~1275 nm (Figure 7) (Equation (8)). The molecular surrounding of the solute fluorophore affects singlet oxygen generation efficiency (Table 2). In polar nonprotic solvent such as acetonitrile, 4Met-BAC exhibits a 20% increase in singlet oxygen production compared to MBAC. In propanol solution, this increase reaches almost 40%. This is due to hydrogen bonding ^3^CT triplet state stabilization. 

The opposite situation is observed in the case of nonpolar solvents such as toluene. In the absence of the solvent stabilization effect, the role of vibrational relaxation increases for 4Met-BAC, and the role of intersystem crossing and fluorescence deactivation pathways diminishes. 

#### 3.1.6. Radical Formation

**Scheme 1:**
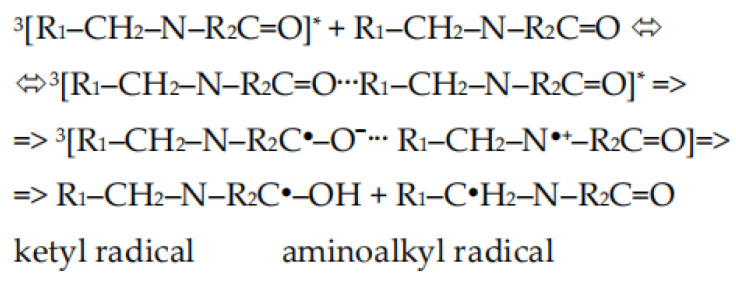
Proposed radical formation mechanism of 4Met-BAC.

It was previously proposed that radical initiation takes place as a result of electron transfer followed by proton transfer (ETPT), resulting in aminoalkyl and ketyl radical formation (Scheme 1) [37]. Unlike aminoalkyl radicals, ketyl radicals hardly initiate the polymerization process [38]. The formation of radical products probably occurs in the electron phototransfer process during interaction between the triplet state and the ground unexcited state of the photosensitizer.

**Figure 8:**
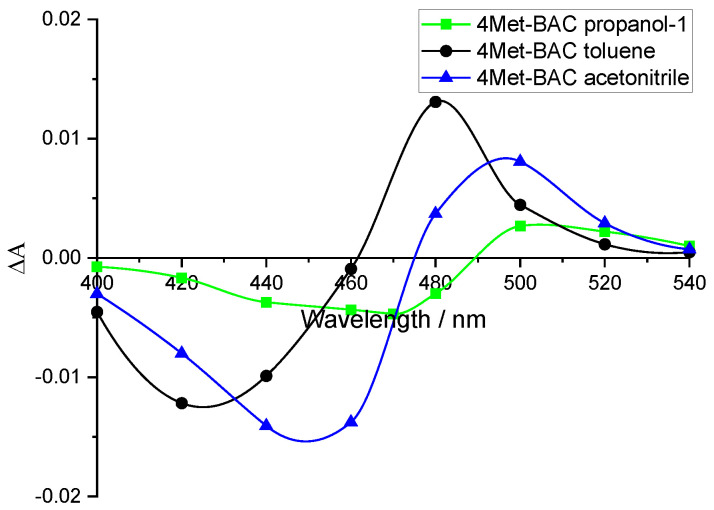
Differential transient absorption spectra of 4Met-BAC (7 × 10^−7^ M) in air-equilibrated solvents (green—propanol-1, black—toluene, blue—acetonitrile) 100 ms after flash.

In order to evaluate the input of radical formation for both 4Met-BAC and MBAC, a series of flash photolysis experiments were conducted using a conventional flash photolysis setup. Photobleaching of a singlet state in the region of 400–460 nm and radical absorption at a longer wavelength (480–540 nm) was registered (Figure 8).

The intensity of this absorption was different in various solvents. More intense radical formation was recorded in toluene and acetonitrile, where the triplet quantum yield was most prominent. 4Met-BAC in propanol-1 showed the least power in terms of radical generation due to low triplet quantum yield and the different nature of the triplet state. Similar results were obtained for MBAC solutions (data not shown).

**Figure 9:**
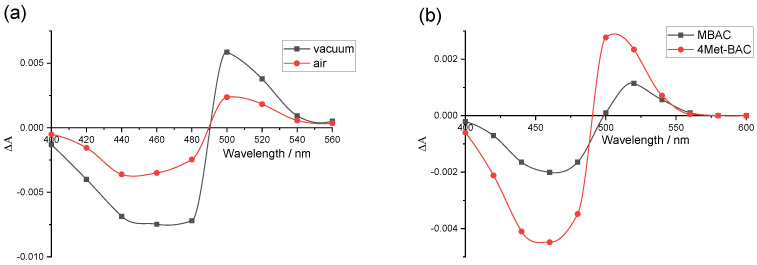
(**a**) Differential transient absorption spectra of 4Met-BAC (7 × 10^−7^ M) in propanol-1 100 ms after flash. (**b**) Differential transient absorption spectra of MBAC (1 × 10^−6^ M) and 4Met-BAC (1 × 10^−6^ M) in air-equilibrated propanol-1 50 ms after flash.

Deaerating the compound solution only resulted in increased signal intensity in the case of propanol-1 (Figure 9a), changing insignificantly in acetonitrile and toluene (data not shown). 

This observation corresponds to the oxygen quenching experiments. In the case of acetonitrile and toluene, the presence of oxygen weakly influenced the intensity of radical formation. Comparing MBAC and 4Met-BAC in terms of radical generation capability, one should note that, in propanol, 4Met-BAC radical absorption was significantly more intense MBAC radical absorption (Figure 9b), which corresponds with the singlet oxygen quantum yield results obtained earlier. 

In this work, photopolymerization takes place in PETA monomer solution, thus providing polar and protic molecular surroundings for the photoinitiator. Taking PI alcohol solution as a model system, we established the enhanced intersystem crossing and radical generation ability of 4Met-BAC.

### 3.2. 3D Structure Fabrication

#### 3.2.1. Influence of DLW Photolithography Parameters on Voxel Size in the TPP Process

**Figure 10:**
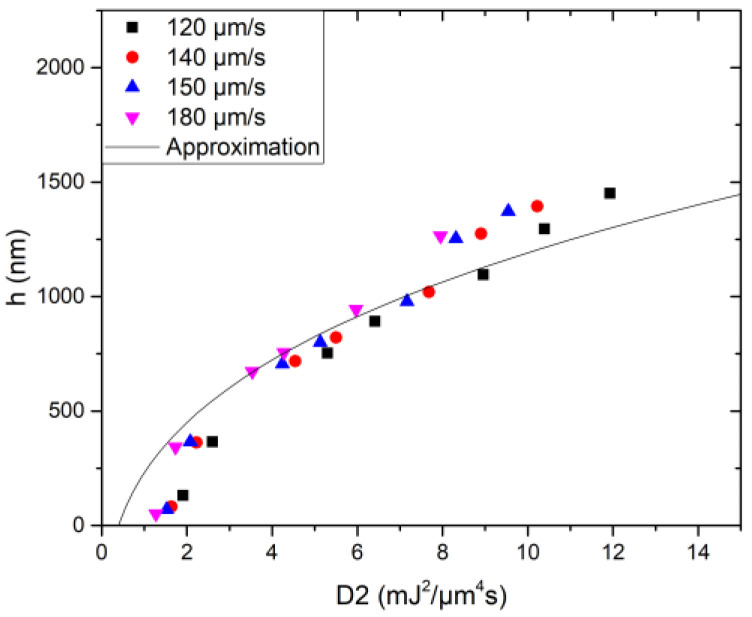
Dependence of volumetric lithography element half-height on the *D*_2_ value.

The influence of DLW photolithography parameters on the physical properties of lithography elements (voxels) was investigated. To obtain the axial voxel size, the AFM method was used (Figure 10). For this study, samples consisting of linear voxel element arrays were fabricated. These were fabricated with different femtosecond laser radiation powers. Variation of the laser radiation power LP was carried out in the range of 3 to 10.5 mW. Within the array, the linear lithography speed V was varied from 120 to 180 µm/s, thus controlling exposure time. The obtained linear voxel elements on the substrate surface did not exceed 1.5 µm in height and had sufficient mechanical stability to preserve morphology during the development process.

The analytical dependence of volumetric lithography element half-height on the DLW photolithography parameters was obtained. This dependence was obtained by studying the two-photon polymerization model under the influence of femtosecond laser radiation. An approximation of the smallness of the radical diffusion rate was applied with respect to the polymerization rate of the exposed volume. The value (9) was used to analyze the results:

(9) $$D_{2}\propto \frac{NA^{3}P^{2}}{\lambda^{3}\upsilon }\propto \frac{P^{2}}{\delta_{Abbe}{}^{3}\upsilon }$$

where $\delta_{Abbe}=\frac{1.22*\lambda }{2NA}=340$ nm is the diffraction limit of Abbe. The quantity of *D_2_* is proportional to the *Q*-index used in [39], which allows for estimation of the influence of lens characteristics on polymerized volume size. The experimental dependence of the half-height of the lithography volume element is approximated by the curve obtained by Equation (10):

(10) $$h\left(D_{2}\right)=\frac{1}{2}*AR_{2}*C*\delta_{Abbe}\sqrt{{\left(\frac{D_{2}}{D_{th}}\right)}^{\frac{2}{3}}-1}-h_{0}$$

where *h* is the measured height of the bulk element (nm), *D_th_* is the threshold dose of radiation (mJ^2^/µm^4^s), *h*_0_ is the height of focus deepening relative to the substrate border (nm), *C* = 0.69 is a constant value, and *AR*_2_ = 2 is the ratio of the voxel’s axial size to its lateral size. When *D_th_* = 0.024 mJ^2^/µm^4^s and *h*_0_ = 450 nm, the mean square deviation of the experimental data from the theoretical approximation does not exceed 250 nm.

#### 3.2.2. Determination of the Degree of Conversion and Reduced Young’s Modulus as Functions of Laser Power

**Figure 11:**
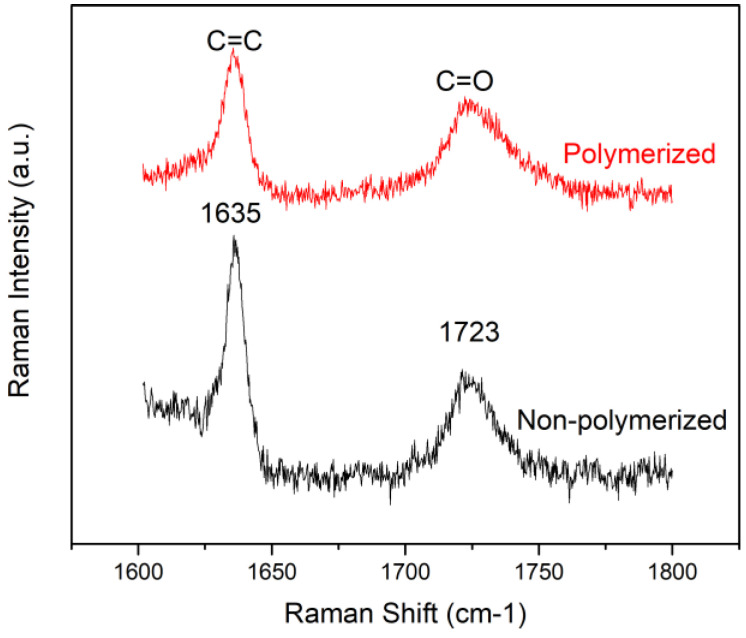
Raman spectrum of the polymerized and non-polymerized photocompositions (4Met-BAC and PETA). The spectra are shifted along the vertical axis for comparison. The black curve corresponds to the spectrum of the non-polymerized photocomposition. Red - for polymerized one.

The degree of conversion, DC, was investigated on polymer structures, which were parallelepipeds with a height of about 10 µm and a square cross-section of 25 µm × 25 µm. The distance between the layers was equal to the half-height of the lithography element (voxel) with the given lithography parameters obtained from previous studies. This was the case in order to control the pumped radiation dose, as well as to increase the height of the structures with fewer layers. All structures were fabricated at a constant hatching distance of 0.2 µm. Linear lithography velocity was constant at 180 µm/s. The structures were fabricated under different laser powers. The value of the conversion degree was calculated using Equation (11) [39,40].

(11) $$DC=\left[1-\frac{A_{C=C}^{poly}/A_{C=O}^{poly}}{A_{C=C}^{resin}/A_{C=O}^{resin}}\right]*100\%$$

where are $A_{C=C}^{poly}$, $A_{C=O}^{poly}$, $A_{C=C}^{resin}$, and $A_{C=O}^{resin}$ represent signal integrals for C=C peaks and C=O peaks of polymerized and non-polymerized resin (Figure 11).

**Figure 12:**
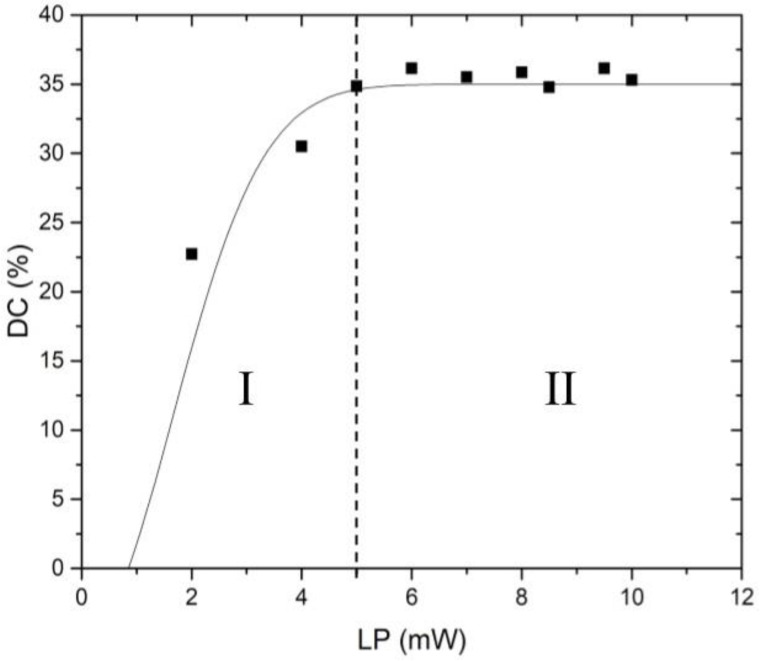
The dependence of conversion degree (DC) on laser power (LP). The solid line shows extrapolation starting from the threshold power of 0.9 mW. The dotted line separates regions I and II, where region I corresponds to conversion degree growth and region II corresponds to conversion degree.

The obtained dependence (Figure 12) illustrates that conversion degree varies directly with laser power in the range of 0 to 5 mW. At laser power higher than 5 mW, conversion degree saturation reaches 34–36%.

Extrapolation of the degree of conversion (Figure 12) was performed using a simple model based on the threshold value [40] and the following Equation (12):

(12) $$DC\left(LP\right)=[C_{1}\left(1-e^{-C_{2}{\left(\frac{LP}{LP_{th}}\right)}^{2}}\right)+C_{0}]*100\%$$

where *C*_2_ = 0.15, *C*_1_ = 0.40, and *C*_0_ = −0.05 represent proportionality coefficients. *LP_th_* = 0.9 mW was the threshold power for radiation used in this photocomposition. Coefficients *C*_1_ and *C*_0_ were chosen so that *C*_1_ + *C*_0_ = *DC_saturation_* and *DC*(*LP_th_*) = 0%. Additionally, coefficient *C*_2_ determines the exit to saturation speed of the conversion degree. Luminescence of the samples was also observed, which was caused by the fact that the photoinitiator is embedded in the polymer chain [24]. This feature of photocomposition can be applied in the study of structures using confocal microscopy [41].

#### 3.2.3. Percolation Formation of Lines

In the process of studies of this photosensitive composition, an unaccounted mechanism for formation of polymerized regions was revealed. Thus, during sequential DLW photolithography of reference line elements (laser irradiation power exceeds the threshold power by an order of magnitude) with a small distance between the lines (order of magnitude of the diffraction limit), the space between them was filled with a thin polymer network, which was later well visualized by SEM. Polymer network formation is related to the diffusion of free monomer radicals and excited photoinitiator molecules in the volume of the photosensitive composition. Long-lived free radicals formed during exposure of the photocomposition to femtosecond laser radiation remain near the polymerized volume in those regions of space, where their concentration is insufficient to form stable polymer chains. Random cross-linking of these radicals is the cause of polymer network formation. The formation mechanism of such polymer networks can be described using the percolation model [42,43,44]. The effect of polymer network formation by this physical mechanism can be applied to the lithography of tiny polymer nanostructures with sizes less than 100 nm [45].

**Figure 13:**
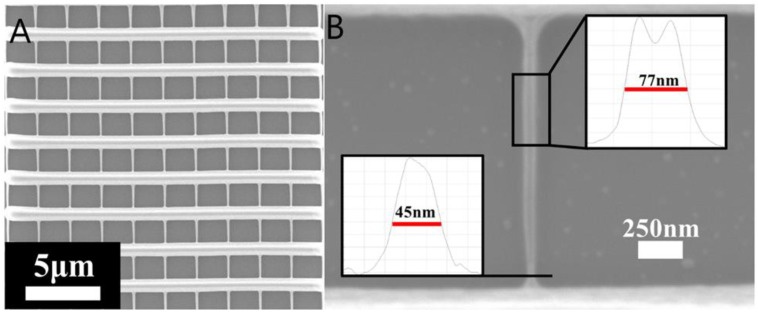
(**A**) SEM image of the structure with percolation lines (vertical). (**B**) SEM image of the percolation line with the smallest size. The insets show the electron scattering intensity profiles for the selected region.

The strategy for controlled formation of “percolation” polymer lines is based on the dynamics of free radical diffusion. Through ponderomotive forces and controlled generation of free radicals in near-threshold lithography, controlled formation of percolation lines/polymerization channels is possible. This strategy was applied to a photosensitive composition based on the 4Met-BAC photoinitiator and PETA monomer. Structures with special geometry were created for experiments on application of the percolation line formation control strategy (Figure 13a). The structures consisted of (1) a reference line array (lithography parameters: 20 mW, 170 µm/s) (horizontal lines on Figure 13a) and (2) a thin/percolation line array (vertical lines on Figure 13a) with a fixed distance between them (2 µm) and a lithography line speed of 170 µm/s. The distance between the reference lines varied from 1 to 4 µm. Thin lines were created with heights above the substrate of 0 to 0.5 µm and with power of 0.25 to 3 mW (threshold power value of 0.9 mW). Fabrication of structures with percolation lines was performed in the following sequence: DLW photolithography of the reference lines array (horizontal lines in Figure 13a,b) was first implemented, followed by thin percolation lines (vertical lines in Figure 13a,b). The sequence of line fabrication with DLW photolithography is important because the free radicals generated in the first step, during polymerization in close proximity to the reference lines, affect the thin-line lithography process in the second step.

As a result of this experiment, thin tiny polymer nanostructures with sizes less than 80 nm (λ/10 of the femtosecond wavelength) were obtained. One of the features of the obtained lines is the asymmetric shape. This type of shape is due to several factors. The lower broadening corresponds to a region with a higher concentration of free monomer radicals and a lower concentration of oxygen. As you move away from the reference lines, the concentration of free radicals decreases and the oxygen concentration reaches the average value in the composition, resulting in thinning of the line. At the same time, new radicals generated by laser radiation are captured in this region. The equilibrium state between the generated radicals and the laser radiation is reached only at a certain distance from the line. This causes a thinning near the beginning of the line (which is thinner than the equilibrium line thickness). When approaching the reference lines, all equilibrium radicals are captured by free radicals near the line and, as a consequence, there is a strong thickening of the created line. 

High repeatability of results is observed for the obtained lines. Among the created lines, line elements with sizes less than λ/10 were obtained, where λ is the wavelength of the laser radiation initiating the TPP process. Thus, the typical line obtained had a thickness at the equilibrium site of 77 nm, and at the narrowest site line thickness reached a value of 45 nm (Figure 13b). Linear element lithography with optimized parameters was performed with 1 mW photopolymerization laser power and a 2 µm gap.

The use of a physical percolation model in the fabrication of lightweight polymer structures makes it possible to create mechanically stable structures with a superresolution.

#### 3.2.4. X-ray Lens Fabrication and Morphology

**Figure 14:**
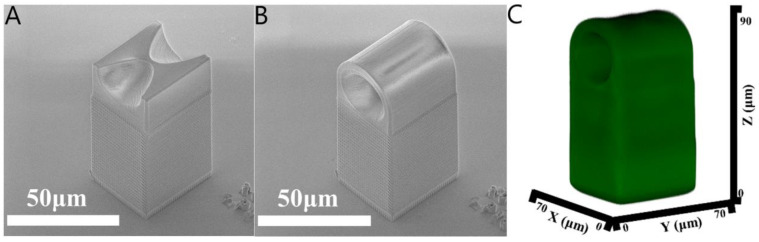
Aspherical lenses for X-rays. (**A**) SEM image of the cut structure. (**B**) SEM image of the structure. (**C**) CLSM image.

The achievement of surfaces in three-dimensional space with the required shape and high values of curvature is in demand for micro-optical element fabrication. including the fabrication of X-ray refractive microlenses. The use of such elements in 3D X-ray refractive microlenses is of interest in the X-ray microscopy field. This type of lens has a large number of advantages, such as quick alignment along the optical axis and the potential use of well-known methods of aberration improvement. The ability to achieve submicron feature size through DLW photolithography, as shown previously, could be used for smooth plastic micro-optics fabrication with nanometer precision. The DLW photolithography method allows one to fabricate 3D structures with arbitrary design. To demonstrate the effectiveness of such an approach, 3D X-ray refractive microlenses with aspherical shape were fabricated (Figure 14). A DLW photolithography algorithm was created in the DeScribe program. To obtain the smoothest surfaces, the structure parameters, the number of layers, the distance between linear elements, the radiation power for two-photon polymerization, and the linear lithography speed were optimized by utilizing the dependence of voxel size on DLW photolithography parameters (Figure 10). Among other things, a laser power above 5 mW was used so that the structure was mechanically stable (Figure 12, region II). Curvation radius of the X-ray lens was 10 µm, and the lens aperture was 24 µm. 

The morphology of this fabricated lens was investigated through Scanning Electron Microscopy (SEM) and Confocal Light Scanning Microscopy (CLSM). CLSM can be used for structures fabricated with DLW because of the strong luminescence of the tetramethacrylic benzylidene cyclopentanone dye integrated into the structure of polymer chains. CLSM and SEM could supplement each other. Analysis of X-ray lens morphology with SEM could provide one with full information about the roughness of surfaces. While using CLSM can provide information about the homogeneity of the physical properties of the X-ray lens polymer.

## 4. Discussion

The solvent effect plays a substantial role in deactivation pathways for MBAC and 4Met-BAC excited states. Protic conditions provide additional stabilization in 4Met-BAC molecules due to hydrogen bond formation with the four methyl methacrylate groups. As a result, fluorescence quantum yield and lifetime increase. Since PI triplet state is CT in nature in protic solvent, it is easily stabilized. Due to additional stabilization of triplet state 4Met-BAC in protic conditions, amplified singlet oxygen quantum yield is observed compared to MBAC. In toluene, the results are opposite: methyl methacrylate substituents provide additional options for vibrational relaxation processes, thus increasing the role of internal conversion and resulting in lower values of fluorescence and singlet oxygen quantum yield for 4Met-BAC. Since the polymerization conditions used in this work in terms of molecular surrounding are similar to PIs in alcohol solution, the latter could be considered as a model system for PI photochemical studies. Under these conditions, the methyl methacrylate derivative (4Met-BAC) shows increased triplet and radical generation ability, which alongside improved solubility in PETA monomer make it a more efficient photoinitiator.

Expanding concentration limits for this initiator in the composition allows for the photosensitivity of the composition to be controlled to a greater extent. Moreover, changes in the concentration of the photoinitiator in the composition do not significantly affect the physical and mechanical properties of the final polymer. These features are provided by chemical similarity between the photoinitiator and the monomer. Additionally, a high overexposure threshold can be associated with the inclusion of the photoinitiator molecules into the polymer chain. Experiments for investigation of the morphological and mechanical properties of polymerized areas during DLW photolithography were carried out with variations in laser power from 0 to 50 mW and varying lithography speeds of 1 to 200 µm/s. Based on this research, an anomalous behavior for laser lithography was determined. When linear lithography speed was in the range of 0 to 50 µm/s, a deviation from the monotonic voxel size increase with decreasing speed was observed. Additionally, lithographical mode increasing the conversion degree with an increase in laser power in the range of 0 to 5 mW was also discovered. A threshold dose value (radiation power of 5 mW at a speed of 180 µm/s) where the studied properties reach saturation values of (35±1) % were identified. These results make it possible to determine optimal parameters for lithography that allow for the production of mechanically stable structures.

## 5. Conclusions

Photochemical properties of a methacrylate benzylidene cyclopentanone derivative were studied. This PI possesses an enhanced ability to generate singlet oxygen and radical intermediates compared to the parent molecule in polar and protic surroundings similar to the PETA monomer environment. The experimental and analytical dependence of the size and elastic properties of voxels on the lithography parameters was established, which is important for the additive method of manufacturing 3D structures. These results make it possible to optimize algorithms for selecting lithography trajectory when creating continuous mechanically stable objects, such as micro-optical elements with high curvature values (including X-ray microlenses). The possibility of using a composition based on the 4Met-BAC photoinitiator to create microstructures for X-ray radiation is also justified due to the possibility of implementing sub-100 nm lithography with a smallest element size of 45 nm.

## Figures and Tables

**Table 1:** Absorption and emission data of 4Met-BAC in different solvents.

Solvent	λ_abs_/nm	λ_flu_/nm	Δυ/cm^−1^	F
Toluene	448	472	1135	0.029
DMSO	474	558	3176	0.841
Propanol-1	473	584	4063	0.779
Acetonitrile	459	544	3404	0.860
Dichloromethane	456	528	2990	0.592
Ethyl acetate	448	514	2866	0.489
Acetone	454	539	3473	0.790

**Table 2:** Photochemical characteristics of studied compounds in different solvents.

Solvent	Compound	Φ_Δ_	Φ_flu_	τ_flu_/ns
Acetonitrile	MBAC	0.14	0.10	0.67
	4Met-BAC	0.17	0.11	0.71
Toluene	MBAC	0.16	0.08	0.30
	4Met-BAC	0.11	0.05	0.24
Propanol-1	MBAC	0.05	0.15	0.46
	4Met-BAC	0.08	0.35	1.16
DMSO	MBAC	-	0.17	0.78
	4Met-BAC	-	0.20	0.84

**Table 3:** 4Met-BAC triplet state characteristics in different conditions.

Solvent	*k*_T_/s^−1^ Ar Purging	*k*_O2_/s^−1^ O_2_ Purging	*k*_diff_/ M^−1^ s^−1^	1/9 *k*_diff_/M^−1^ s^−1^	*k*_q_/M^−1^ s^−1^
Acetonitrile	8.0 × 10^6^	4.5 × 10^7^	1.9 × 10^10^	2.1 × 10^9^	3.1 × 10^9^
Propanol	5.2 × 10^5^	2.0 × 10^7^	3.4 × 10^9^	3.7 × 10^8^	4.5 × 10^8^

## Data Availability

Not applicable.

## Supplementary Materials

The following supporting information can be downloaded at: https://www.mdpi.com/article/10.3390/polym15010071/s1, Figure S1: HNMR spectrum for 4Met-BAC; Figure S2: CNMR spectrum for 4Met-BAC.
