# Supplementary figures and images for: Benzylidene Cyclopentanone Derivative Photoinitiator for Two-Photon Photopolymerization-Photochemistry and 3D Structures Fabrication for X-ray Application
Source: Polymers (Basel). 2022 Dec 24;15(1):71. doi: 10.3390/polym15010071 (PMC9823431; doi:10.3390/polym15010071)

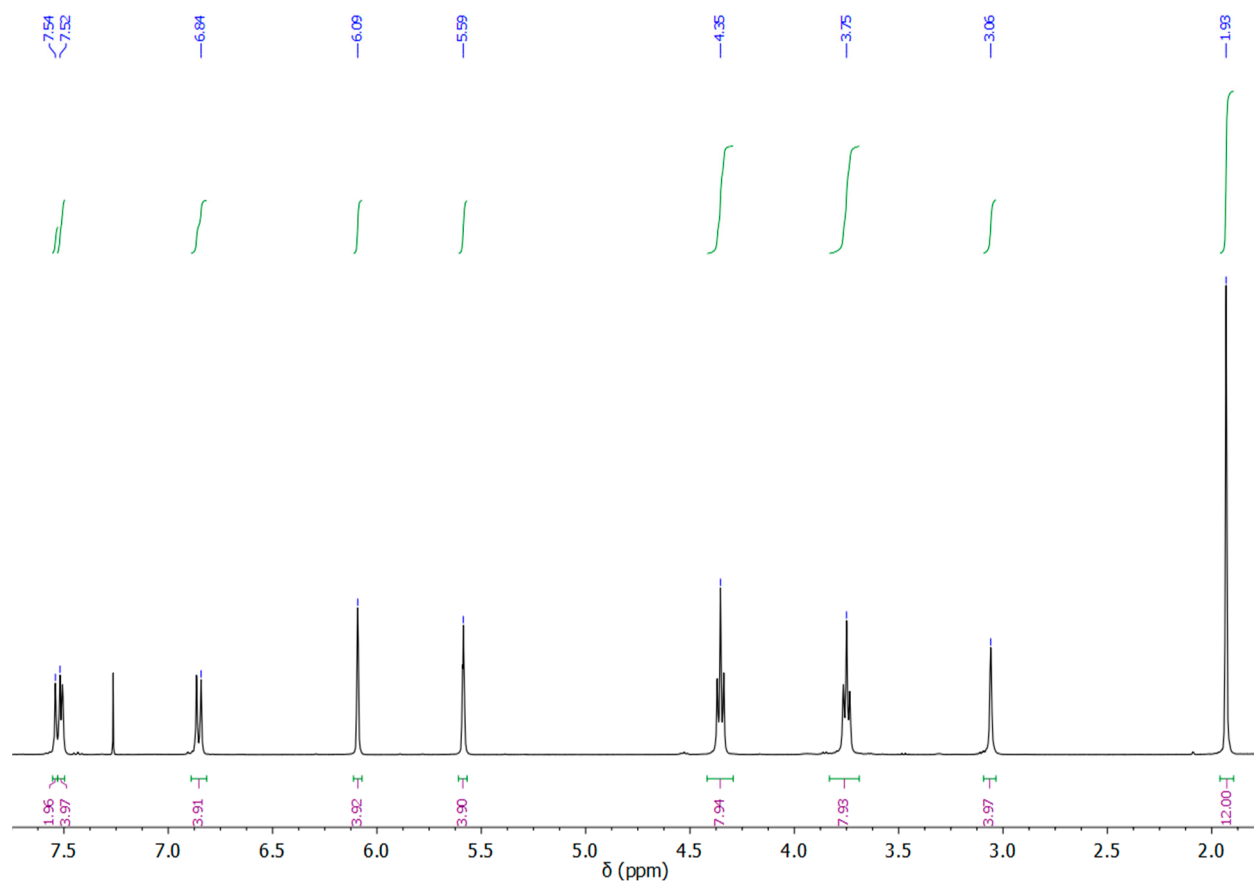

Figure S1  $^1\text{H}$  NMR spectrum for 4Met-BAC.

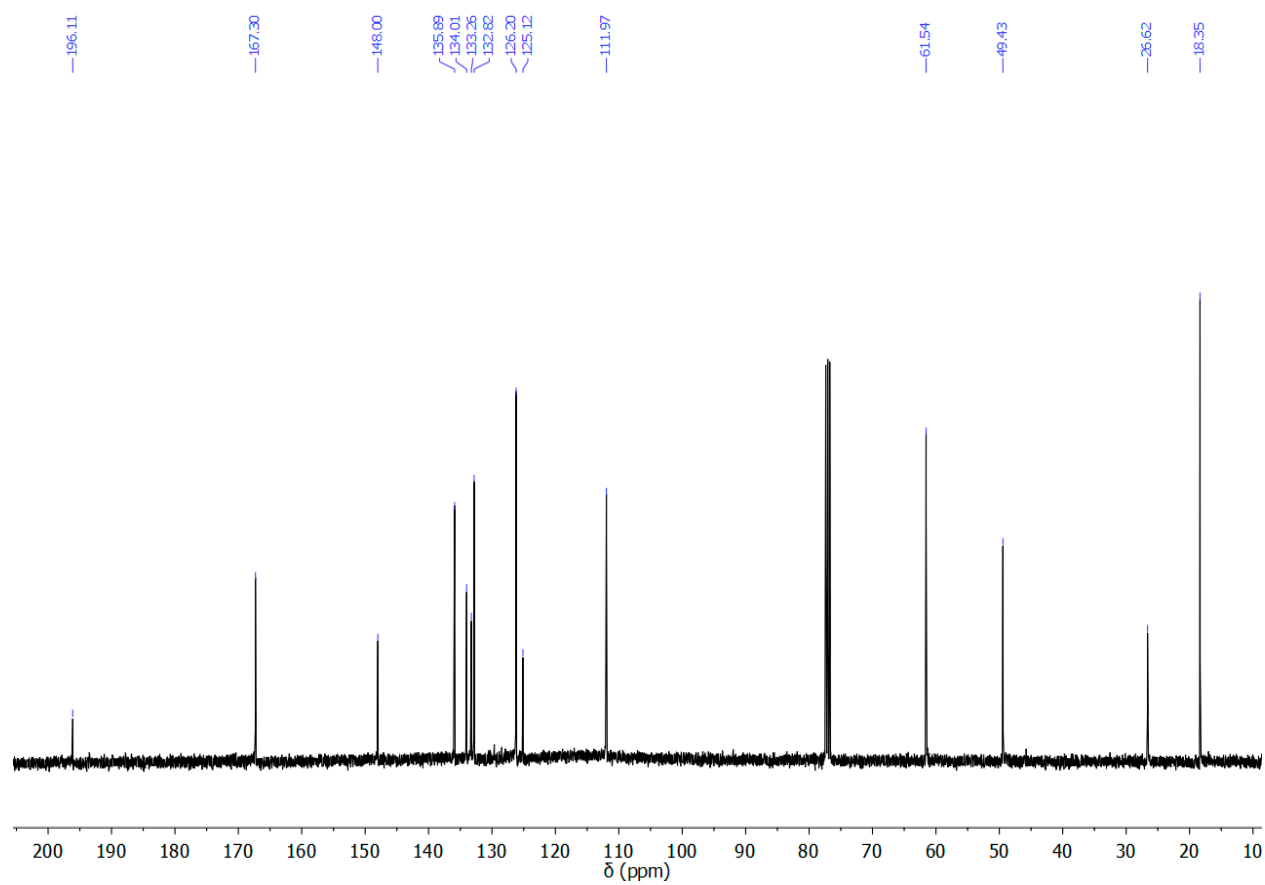

Figure S2 CNMR spectrum for 4Met-BAC.

Supplement: Supplementary file 1 [file polymers-15-00071-s001.zip › polymers-2120666-supplementary.pdf]
